# Alpha‐internexin is detectable in cerebrospinal fluid – an analogue to neurofilament light?

**DOI:** 10.1002/alz.092208

**Published:** 2025-01-09

**Authors:** Francisco J Meda, Camilla Johansson, Karin Palm, Gunnar Brinkmalm, Ulf Andreasson, Kaj Blennow, Hlin Kvartsberg, Henrik Zetterberg

**Affiliations:** ^1^ Department of Psychiatry and Neurochemistry, Institute of Neuroscience and Physiology, the Sahlgrenska Academy at the University of Gothenburg, Mölndal Sweden; ^2^ Clinical Neurochemistry Laboratory, Sahlgrenska University Hospital, Mölndal Sweden; ^3^ Department of Psychiatry and Neurochemistry, Institute of Neuroscience and Physiology, The Sahlgrenska Academy, University of Gothenburg, Mölndal, Gothenburg Sweden; ^4^ Hong Kong Center for Neurodegenerative Diseases, Hong Kong China; ^5^ Sahlgrenska University Hospital, Gothenburg Sweden; ^6^ Department of Neurodegenerative Disease and UK Dementia Research Institute, UCL Institute of Neurology, Queen Square, London UK; ^7^ Wisconsin Alzheimer’s Disease Research Center, University of Wisconsin School of Medicine and Public Health, Madison, WI USA; ^8^ UK Dementia Research Institute, University College London, London UK; ^9^ Department of Psychiatry and Neurochemistry, Institute of Neuroscience and Physiology, The Sahlgrenska Academy at the University of Gothenburg, Mölndal Sweden

## Abstract

**Background:**

Alpha‐internexin (AINX) is a type IV intermediate filament alongside the neurofilament triplet proteins. Although AINX is mostly expressed during the development of the brain, it is still present in mature neurons and reported to be involved in axonal outgrowth. Because of a lack of previously published studies quantifying AINX in cerebrospinal fluid (CSF), and its high sequence similarity with other neurofilaments, the objective of this project was to specifically detect AINX via antibody‐based techniques and assess its biomarker capabilities.

**Method:**

AINX in‐house antibodies (referred to as B15 and Ina1), alongside commercial antibodies (PA5‐95368 and MA5‐32809) were used to detect AINX in CSF and brain using ELISA and Simoa platforms. A recombinant AINX protein (AINX 89‐499) produced in‐house was used as positive control and calibrator. All antibodies were conjugated on beads, coated on microtiter plates, or biotinylated following standard protocols for usage on both platforms. Immunoprecipitation followed by mass spectrometry (IP‐MS), was also performed to evaluate antibody binding and specificity.

**Result:**

In ELISA, all antibody combinations (capture/detector) were able to detect recombinant protein, with B15, Ina1 and PA5‐95368 providing the most effective combinations. While we were able to detect AINX in SDS brain extracts, only a few CSF samples generated any signal using ELISA. The low signals in CSF indicated that the sensitivity of ELISA might be a limiting factor. Therefore, we moved to the ultrasensitive Simoa platform. Here, we were able to show that AINX is detectable in CSF at low concentrations (1‐10pg/mL), with its increase correlating with CSF neurofilament light levels. IP‐MS results on SDS brain extracts revealed high sequence coverage of AINX for all three antibodies (90% for PA5‐95368, 56% for B15 and 85% for Ina1) thus confirming antibody specificity.

**Conclusion:**

All antibodies tested seem to be very suitable for immunoassay development and AINX is present in CSF at low abundance, with the Simoa platform being able to detect it due to its high sensitivity. Future work will focus on evaluating the biomarker potential of AINX in different neurodegenerative diseases and acute conditions such as stroke or traumatic brain injury.

